# Effect of physicochemical parameters on *Anopheles* and *Culex* mosquito larvae abundance in different breeding sites in a rural setting of Muheza, Tanzania

**DOI:** 10.1186/s13071-017-2238-x

**Published:** 2017-06-24

**Authors:** Basiliana Emidi, William N. Kisinza, Bruno P. Mmbando, Robert Malima, Franklin W. Mosha

**Affiliations:** 1 Kilimanjaro Christian Medical University College, P.O. Box 2240, Moshi, Tanzania; 2 National Institute for Medical Research, Headquarters, P.O. Box 9653, Dar es Salaam, Tanzania; 3 National Institute for Medical Research, Amani Centre, P.O. Box 81, Muheza, Tanzania; 4 National Institute for Medical Research, Tanga Centre, P.O. Box 5004, Tanga, Tanzania

**Keywords:** *Anopheles*, *Culex*, Mosquitoes, Breeding sites, Physicochemical parameters, Muheza, Tanzania

## Abstract

**Background:**

Malaria and lymphatic filariasis (LF) are diseases of great public health important in East Africa. Malaria is transmitted by *Anopheles* while LF is transmitted by both *Anopheles* and *Culex* mosquitoes. There is limited evidence on the effects of physicochemical parameters on these mosquitoes in rural settings of Tanzania. This study aimed at assessing the effects of physicochemical parameters on *Anopheles* and *Culex* larvae abundance in different breeding sites in a rural setting of Muheza district**.**

**Methods:**

A cross-sectional study was conducted in 13 villages in Muheza district, between December 2015 and May 2016. Mosquito larvae were sampled using standard dipping techniques. Physicochemical parameters were measured by a Multi-parameter pH meter in different mosquito breeding sites. Mosquito larvae and pupa densities were compared between the ≤33th (lower) and ≥67th (upper) percentiles of physicochemical parameters. *An. gambiae* (*s.l.*) and *An. funestus* were identified by polymerase chain reaction (PCR) to reveal their sibling species.

**Results:**

Abundance of *Anopheles* larvae was significantly higher (76.6%) than *Culex* (66.9%) *χ* ^2^ = 5.73, *df =* 1, *P* = 0.017). The presence of late instars of *Anopheles* was significantly higher (78.2%) than that of *Culex* (64.5%) (*χ* ^2^ = 0.984, *df* = 1, *P* = 0.017). A model adjusted for larval stage showed that the likelihood of finding *Culex* larvae was lower by 38.2% (95% CI: 16.9–54.1, *df* = 1, *P* = 0.001) compared to *Anopheles.* Upper percentiles of salinity (OR = 7.05; 95% CI: 1.19–41.88, *P* = 0.032) and conductivity (OR = 5.47; 95% CI: 1.01–29.67, *P* = 0.056) were significantly associated with the presence and with increased density of *Anopheles* larvae. PCR results showed that, within the *gambiae* complex, 53.3% (*n* = 136) were *An. gambiae* (*s.s*.) and 46.7% (*n* = 119) were *An. arabiensis.* In *An. funestus* group, 91.1% (*n* = 41) were *An. funestus* (*s.s*.)*,* 4.4% (*n* = 2) were *An. rivulorum*, 2.2% (*n* = 1) were *An. leesoni* and 2.2% (*n* = 1) were *An. parensis.*

**Conclusion:**

High salinity and conductivity were significantly associated with increased density of *Anopheles* larvae. To our knowledge, this is the first study to demonstrate the occurrence of *Anopheles* larvae in polluted breeding sites in rural settings of Tanzania. The study has found both *Anopheles* and *Culex* mosquito larvae co-existed in breeding sites. The possible reasons for tolerance to a higher level of physicochemical parameters among *Anopheles* mosquitoes need to be ascertained.

## Background

Malaria and lymphatic filariasis are diseases of significant public health importance in East Africa [1–3]. Malaria is transmitted by anopheline mosquitoes, while lymphatic filariasis is transmitted by both *Anopheles* and *Culex* mosquitoes [2, 4, 5]*.* These vectors breed in various habitats [6, 7]. For instance, whereas *An. gambiae* (*s.l.*) breed in small, open, sunlit, fresh water bodies, *An. funestus* normally breed in water bodies with emergent vegetation such as swamps and rice fields. *Culex quinquefasciatus* breed in polluted water habitats such as pit latrines, soak pits, cesspits and open sewage systems [6, 8].

The breeding sites of these mosquitoes are sometimes contaminated with pollutants from various sources such as sewage and fertilisers from agricultural fields [9, 10]. Physicochemical characteristics of mosquito breeding sites may have some effect on mosquito vectors oviposition, survival and spatial distribution [9]. Physicochemical parameters such as temperature, salinity, conductivity, total dissolved solids (tds) and pH have a significant influence on the occurrence and larval abundance among mosquito species [8, 11, 12]. High levels of conductivity may be due to the application of agricultural pesticides and herbicides [9].

Climatic factors such as moisture index and temperature also have strong effects on the distribution and abundance of malaria vectors [13–16]. Mosquito vector distribution is to a large extent influenced by climatic conditions and species habits across the globe [6, 17, 18]. Their population densities vary with seasons due to fluctuating availability of favourable breeding sites [19]. Availability, distribution and abundance of mosquitoes depend on types of breeding sites including water surface area and other biological factors [20, 21]. The density of adult mosquitoes is determined by the number and productivity of larval habitats and their proximity to human hosts where they can obtain a blood meal [22]. A better understanding of breeding behavioural patterns among mosquito populations is one of the key elements for reaching the goal of malaria and lymphatic filariasis elimination and eradication [7, 23]. While various factors affecting the breeding of *Culex* and *Anopheles* mosquitoes have been documented, there is limited evidence on the effects of physicochemical parameters on these mosquito vectors in sub-Saharan Africa. This study, therefore, aimed at assessing the effects of physicochemical parameters on *Anopheles* and *Culex* larvae abundance in different breeding sites in a rural setting of Muheza district, Tanzania.

## Methods

### Study area

**Fig. 1:**
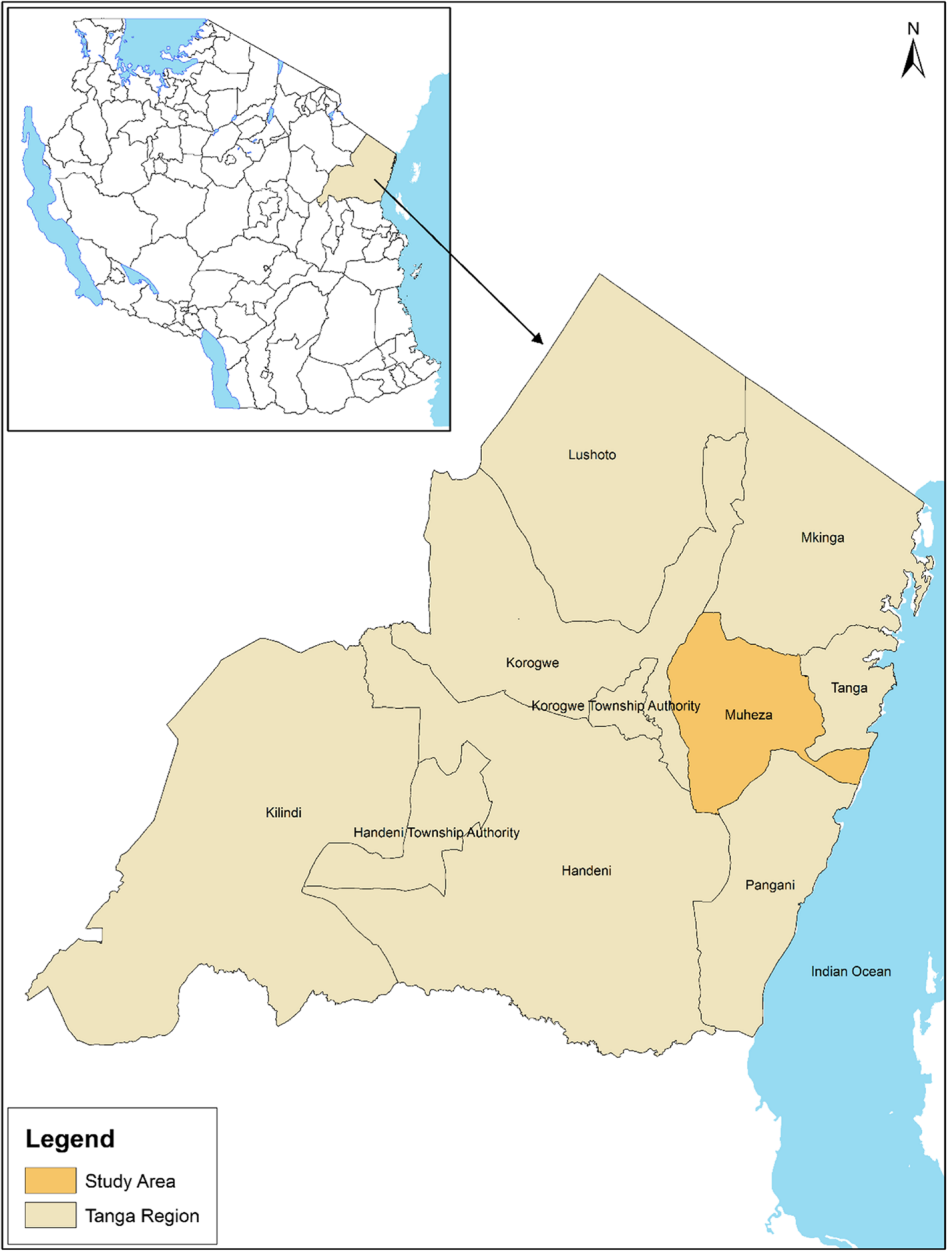
Map of Tanga region showing Muheza District

This study was conducted in Muheza district along the north-eastern coast of Tanzania. Muheza is 4922 km^2^, and it is located 5°10′S, 38°46′E. The district stretches from a coastal plain at sea level to the Usambara Mountains at an elevation of 1050 m above sea level (Fig. 1). According to the 2012 Population and Housing Census, the district had a total of 204,461 people, of whom 100,843 (49.3%) were males while 103,618 (50.7%) were females [24]. The climate is tropical, with dense rainforest cover over the Usambara mountain ranges with annual rainfall 1000–2000 mm. Muheza district is mainly inhabited by subsistence farmers, where rain-fed rice, maize, oranges and vegetables are the main crops. Administratively, Muheza district is divided into 6 divisions comprised of 35 wards with 175 villages [24].

The dominant mosquito species include *An. gambiae* (*s.l.*), *An. funestus* and *Culex quinquefasciatus.* These vectors are most abundant during the rainy seasons, which include long rains in April to September and short rains in December to January.

### Study design and sites

**Fig. 2:**
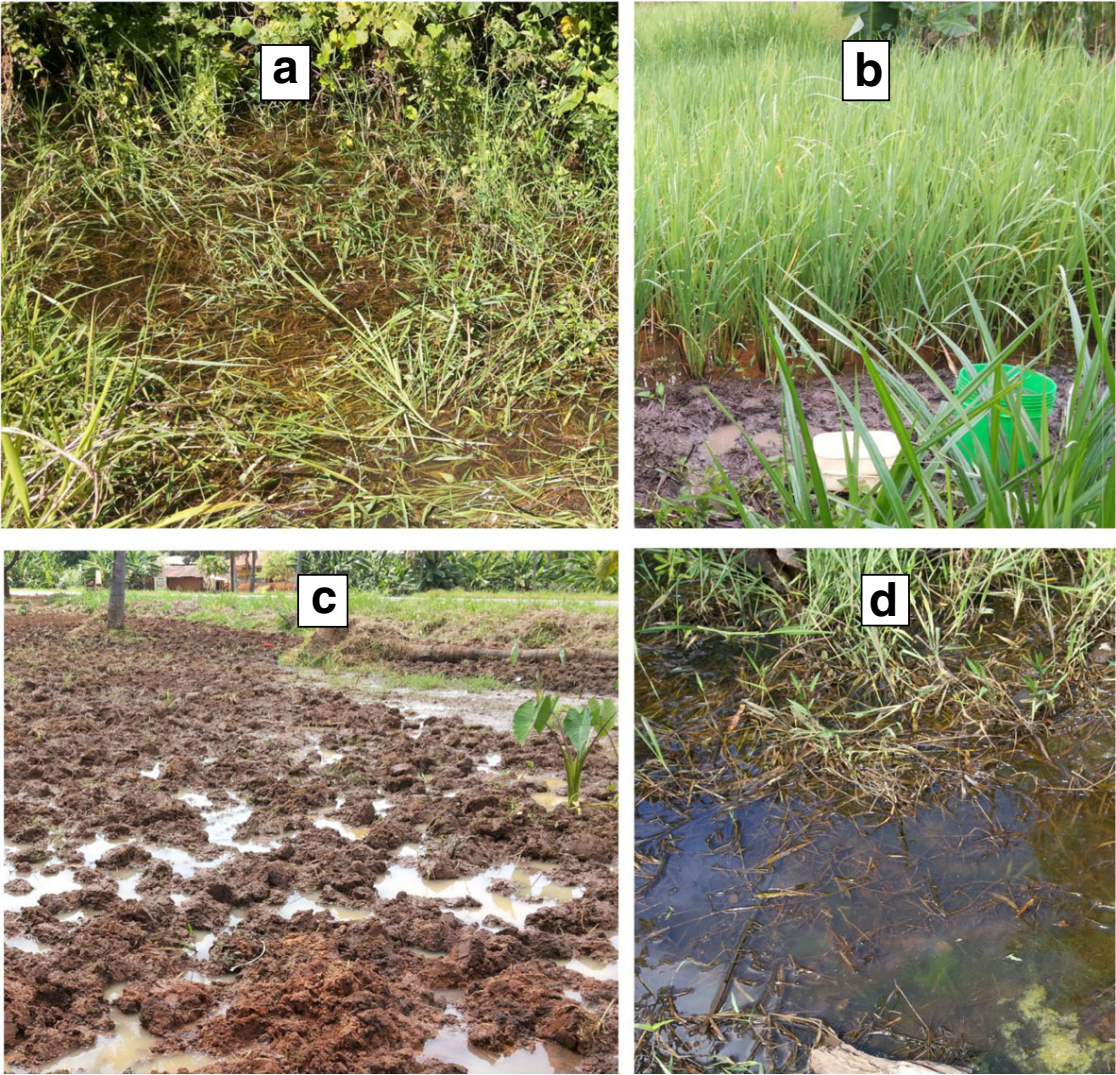
Mosquito breeding sites. **a** Swamp. **b** Rice-fields. **c** Hoof-prints. **d** Rainwater collections

This was a cross-sectional study that involved 13 villages, which were each visited once between December 2015 and May 2016. The selected villages included Kicheba, Mamboleo, Mkuzi-2, Bwembwera, Kibaoni-2, Mamboleo-Lusanga, Mbaramo, Mianga, Mkurumilo, Pangamlima, Songakibaoni-2, Umba and Zeneti. The villages were surveyed for mosquito breeding sites and their physicochemical parameters. During the survey, a total of 31 sampling points with eight types of breeding sites (rainwater collections, rice fields, animal hoof-prints, swamps, roadside water collections and road potholes, pond water reservoirs, small ponds and water collections on the sides of the bridge) were surveyed (Fig. 2). During analysis, each village’s measurements of physicochemical parameters were to some extent expected to reflect the season of sampling and were independent of seasons.

### Mosquito larvae sampling and rearing

To obtain adult mosquitoes for morphological identification, larvae were sampled using a standard dipping technique from various breeding sites in each village using two types of dipper. One was the standard dipper described by WHO [7] and the other was an improvised dipper described by Emidi et al. [25] which is suitable for small breeding sites such as animal hoof prints. A total of 50 to 100 mosquito larvae were collected per village and reared at the insectary for morphological identification of emerging adults. The number of mosquito larvae ranged from 50 to 100 among the villages sampled. Only 50 larvae were collected from Mkurumilo and Umba villages while 100 larvae were obtained from rest of the villages. These larvae were pooled per village irrespective of their breeding sites. Emerging adult mosquitoes were morphologically identified using a morphological key by Gillies & DeMeillon [26] for *Anopheles* species and Edwards [27] for culicines. However, adult *An. gambiae* (*s.l.*) and *An. funestus* were preserved in silica gel for further molecular identification of their sibling species by quantitative real-time polymerase chain reaction (qPCR).

### Molecular identification of sibling species of *An. gambiae* (*s.l.*) and *An. funestus* group

Out of 1250 emerged adult mosquitoes of *An. gambiae* (*s.l.*) and *An. funestus* group, a total of 320 mosquitoes were identified by qPCR as described in [28] to reveal the identity of the sibling species.

### Collection of physicochemical parameters data

Physicochemical parameters (temperature, pH, conductivity, tds and salinity) were measured using a multiparameter pH meter (Eutech PCSTEST35-01X441506/Oakton 35,425–10, Vernon Hills, Illinois, 60,061, USA) based on guidelines provided by the manufacturer. The multiparameter pH meter was calibrated before use. A total of four replicate measurements were recorded per breeding site. All mosquito breeding sites encountered were sampled. A total of 31 sampling points was surveyed, and variations in the frequency of sampling per village depended on the availability of breeding sites.

### Data analysis

Data were analysed in Microsoft Excel and the Stata 13 and R 3.3.0 statistical packages. Categorical data were summarised by cross tabulation and compared using chi-square. Continuous variables were summarised using mean and standard deviation and compared using Student’s t-test for independent samples. Mosquito larvae densities were highly skewed and so were square root-transformed. Presence/absence and density of the larvae and pupae in the breeding sites were modelled using generalised estimating equation (GEE) for binary and continuous data, respectively. The GEE model was chosen because data were correlated between breeding sites and in replications. The binary GEE model was used to determine the importance of different levels of the physicochemical parameters on the presence of mosquito larvae or pupae, while the linear GEE was used to determine the association between the transformed larvae and pupae densities and physiochemical parameters. Both, univariate and multivariate models were fitted, and estimates were reported with robust 95% confidence intervals (95% CI). Physicochemical parameters were categorised into three groups; ≤ 33rd, 33–66th and ≥67th percentiles to increase the power by increasing the variability of larvae and pupae densities by the group of physicochemical parameters. This categorization was selected after the model fitting physiochemical parameters as a continuous variable, revealed low power in order to attain the significant levels of the coefficients. The comparisons were made between the first and the last groups, which in this paper will be referred to as lower and upper percentiles, respectively.

Mosquito breeding sites with comparatively smaller variations in mean values of the physicochemical parameters were grouped together to reduce the number of breeding sites. A total of six breeding site types were obtained following pooling of mosquito breeding sites: (i) rain water collections; (ii) road-side water collections and road potholes; (iii) rice fields and animal hoof prints; (iv) swampy areas; (v) pond water reservoirs; and (vi) small ponds and water collections on the sides of bridges. In all analyses, a *P*-value of <0.05 was considered statistically significant. During analysis, each village measurements of physicochemical parameters were independent of seasons and mosquito breeding site type.

## Results

### Dominant breeding site types and their frequency in surveyed villages

All breeding sites encountered during the survey were sampled. Among the mosquito breeding sites sampled, the ones with high frequencies of occurrence were described as “dominant breeding sites”. Out of 31 encountered sampling points, 10 (32.3%) were swamped, and 7 (22.7%) were rainwater collection ponds; these constituted the dominant breeding sites. Regarding the number of habitats from each village; Mkuzi, Mamboleo and Kicheba villages had four breeding sites each; Mbaramo had one while the rest had had two breeding sites each. The types and frequencies of breeding sites per village are shown in Table 1. During analysis, the breeding sites with minimal difference in physicochemical parameters were combined as described in the data analysis section.

**Table 1:** Types and frequency of breeding sites per village

		Breeding site type code									Frequency of breeding sites per village
	Villages	1		3	4	5	6	7	11	13	
1	Mkuzi	4		0	0	0	0	0	0	0	4
2	Kicheba	0		0	0	0	0	3	1	0	4
3	Umba	0		0	0	2	0	0	0	0	2
4	Kibaoni-2	1		0	0	0	1	0	0	0	2
5	Zeneti	0		1	1	0	0	0	0	0	2
6	Mamboleo-Lusanga	0		0	0	2	0	0	0	0	2
7	Bwembwera	0		0	1	0	1	0	0	0	2
8	Mbaramo	0		0	0	1	0	0	0	0	1
9	Songakibaoni-2	1		0	0	1	0	0	0	0	2
10	Mkurumilo	0		0	0	0	1	0	0	1	2
11	Mianga	0		0	0	1	0	0	0	1	2
12	Pangamlima	1		0	0	0	1	0	0	0	2
13	Mamboleo	0		1	0	3	0	0	0	0	4
	Total types of breeding sites	7		2	2	10	4	3	1	2	31

*Key*: 1, rain water collections; 3, rice fields; 4, animal hoof prints; 5, swampy areas; 6, road side water collections and road potholes; 7, pond water reservoirs; 11, small ponds; 13, water collections on sides of the bridges

Temporary breeding sites can contain water for several weeks but normally dry up after the rainy season while permanent breeding sites can contain water even after the rainy season. In the context of this study, temporary breeding sites were rainwater collections, roadside water collections and tyre prints, rice fields and animal hoof prints and ponds/small ponds and road potholes while permanent breeding sites were swampy areas and pond water reservoirs. The main sources of water for these breeding sites are rainfall.

### Mosquito mean larvae and pupae abundance

*Anopheles* and *Culex* mosquito species were found to co-exist in the breeding sites. The overall mean abundance of larval stages 1 and 2 (early instars) of *Anopheles* was 11.2 larvae per dipper while that of *Culex* was 11.6 and the difference was not significant (*t*_(60)_ = -0.08, *P* = 0.9). The mean abundance for larval stages 3 and 4 (late instars) of *Anopheles* was 8.2 larvae per dipper while that of *Culex* was 10.3, and these were not significantly different (*t*_(60)_ = -0.75, *P* = 0.45). There was no significant difference in the mean density of pupae across the breeding site types (Kruskal-Wallis test, *χ* ^2^ = 3.53, *df* = 5, *P* = 0.619).

There were more immature stages of *Anopheles* than *Culex* in the sites surveyed; the probability of finding *Anopheles* larvae was 76.6%, while that of finding *Culex* was 66.9%, (*χ* ^2^ = 5.73, *df* = 1, *P* = 0.017). The occurrence of early instars was similar between the *Anopheles* (75%) and *Culex* (69.3%), (*χ* ^2^ = 0.984, *df* = 1, *P* = 0.321), while that of late instars, was significantly higher in *Anopheles* (78.2%) than in *Culex* (64.5%), (*χ* ^2^ = 5.70, *df* = 1, *P* = 0.017). The GEE model adjusted for larval stage showed that the likelihood of finding *Culex* larvae was lower by 38.2% (95% CI: 16.9–54.1, *df* = 1, *P* = 0.001) compared to *Anopheles.*

### Identification of adult mosquito, and *An. gambiae (s.l.)* and *An. funestus* sibling species

Two mosquito genera obtained from reared larvae were *Anopheles* and *Culex*. Upon morphological identification of adult mosquitoes, several species of mosquitoes were obtained. The genus *Anopheles* was comprised of *An. gambiae* (*s.l.*), *An. funestus* group, *An. coustani* and *An. maculipalpis* while the genus *Culex* comprised of *Cx. quinquefasciatus* and *Cx. cinereus* species. The male/female ratio of emerged mosquitoes was almost equal to 1:1. *Anopheles funestus* mosquitoes were collected from Zeneti village only. Our target mosquito species were *An. gambiae* (*s.l.*)*, An. funestus* group and *Cx. quinquefasciatus.* Other species of mosquitoes obtained such as *An. coustani, An. maculipalpis* and *Cx. cinereus* were discarded after sorting.

Regarding the *An. gambiae* (*s.l.*) and *An. funestus* sibling species, a total of 320 *Anopheles* mosquitoes were further identified to sibling species by qPCR. Results from PCR have shown that, within the *An. gambiae* complex, only two sibling species were identified. Out of 255 *An. gambiae complex*, 53.3% (*n* = 136) were *An. gambiae* (*s.s.*) and 46.7% (*n* = 119) were *An. arabiensis.* There were no *An. merus* identified. In addition, four sibling species within the *An. funestus* group were identified. Out of 45 *An. funestus* group, 91.1% (*n* = 41) were *An. funestus* (*s.s.*)*,* 4.4% (*n* = 2) were *An. rivulorum,* 2.2% (*n* = 1) were *An. leesoni* and 2.2% (*n* = 1) were *An. parensis.* None amplified samples accounted for 6.7% (*n* = 20).

### Physicochemical parameters of different breeding sites

Table 2, shows the categorization of physicochemical parameters measured in the mosquito breeding sites. Lower percentile of salinity ranged from 107.0–245.0 ppm while higher percentiles ranged from 576.9–1190.0 ppm. Other percentiles and variables are as shown in Table 2. The higher percentiles in our model were more stringent than those reported from other categorizations [29].

**Table 2:** Distribution of levels of physicochemical parameter percentiles when compared to standard categories

Parameter	Percentile	Range (min-max)	Standard range [29]	Description [29]
Salinity (ppm)	≤ 33%	107.0–245.0	< 500	Fresh water
	≥ 67%	576.9–1190.0	≥ 500	Polluted water
Temperature (°C)	≤ 33%	24.5–30.4	< 35	Normal temperature
	≥ 67%	36.6–42.8	≥ 35	Hot
Total dissolved solids (ppm)	≤ 33%	131.0–289.0	< 400	Mineral springs
	≥ 67%	701.0–1450.0	≥ 400	High contamination
Conductivity (μS)	≤ 33%	200.0–411.0	< 800	Domestic water
	≥ 67%	1007.0–1970.0	≥ 800	Highly polluted
pH	≤ 33%	7.0–7.9	7	Neutral
	≥ 67%	8.4–11.0	11	Weak basic

### Effect of physicochemical parameters on the presence or absence of mosquito larvae

Results from GEE model for the parameters associated with presence or absence of mosquito larvae showed that upper percentiles of salinity (OR = 7.05; 95% CI: 1.19–41.88, *P* = 0.032) and conductivity (OR = 5.47; 95% CI: 1.01–29.67, *P* = 0.056) were strongly associated with the presence of *Anopheles* larvae. Although not statistically significant, high tds was associated with the presence of *Anopheles* larvae and pupae. None of the parameters was found to be significantly associated with the presence of *Culex* larvae*.* Additionally, breeding site types had no significant effect on the presence of mosquito larvae and pupae (Table 3).

**Table 3:** Association between presence of mosquito larvae and pupae densities with breeding site type and physicochemical parameters

Parameter	*Anopheles* larvae	*Culex* larvae	Pupae (*Culex* and *Anopheles*)
	OR (95% CI); *P*-value	OR (95% CI); *P*-value	OR (95% CI); *P*-value
Breeding site type			
(i) Rain water collections	1	1	1
(ii) Road side water collections and road potholes	0.83 (0.11–6.38); 0.858	2.12 (0.17–26.11); 0.559	0.67 (0.06–7.54); 0.743
(iii) Rice fields and animal hoof prints	0.68 (0.05–10.0); 0.781	0.18 (0.02–1.68); 0.133	1.2 (0.07–20.56); 0.900
(iv) Swampy areas	0 .99 (0.17–5.58); 0.988	0 .97 (0.15–6.24); 0.975	1.2 (0.15–9.29); 0.861
(v) Pond water reservoirs	0.46 (0.04–5.02); 0.528	0.22 (0.03–1.84); 0.161	0.29 (0.03–2.65); 0.270
(vi) Small ponds and water collections on the sides of the bridges	0 .10 (0.01–1.70); 0.110	0.30 (0.03–3.47); 0.337	0.2 (0.01–3.84); 0.286
Physicochemical parameter			
Salinity (ppm)	7.05 (1.19–41.88); 0.032^a^	1.22 (0.3–4.93); 0.78	3.51 (0.73–16.83); 0.117
Temperature (°C)	2.65 (0.59–11.73); 0.197	0.4 (0.11–1.48); 0.17	0.55 (0.12–2.67); 0.456
Total dissolved solids (ppm)	4.20 (0.71–24.87); 0.113	0.79 (0.2–3.08); 0.73	3.23 (0.66–15.86); 0.149
Conductivity (μS)	5.47 (1.01–29.67); 0.057^a^	0.76 (0.2–2.83); 0.68	2.84 (0.62–13.06); 0.180
pH	0.45 (0.11–1.85); 0.218	0.66 (0.19–2.2); 0.49	0.48 (0.174–1.31); 0.153

*Abbreviation*: *OR* odds ratio

^a^ Statistically significant

### Association between physiochemical parameters on *Anopheles* and *Culex* mosquito larvae, and pupa densities

Table 4 shows the means ± standard deviations of physicochemical parameters in the six categories of various mosquito breeding sites. Total dissolved solids (tds) were highest in pond water reservoirs (990 ± 550.7 ppm) and lowest in the small ponds and water collection on bridge sides (249.3 ± 103.9). The pH in all breeding sites was weak basic. Salinity and conductivity were recorded highest in the pond water reservoirs with 809.2 ± 453.9 ppm and 1336 ± 738.1 μS, respectively.

**Table 4:** Distribution of mean levels of physicochemical parameters and mosquito larvae and pupae densities by breeding site type

Breeding site type categories (*n*)	Temperature (mean ± SD)	Tds (mean ± SD)	pH (mean ± SD)	Salinity (mean ± SD)	Conductivity (mean ± SD)	*Anopheles* (mean ± SD)	*Culex* (mean ± SD)	Pupae (mean ± SD)
(i) Rain water collections (7)	33.0 ± 4.7	558.3 ± 260.8	8.0 ± 0.5	464.8 ± 248.8	653.9 ± 311.5	11.0 ± 9.7	15.6 ± 13.0	8.0 ± 7.1
(ii) Road side water collections and road potholes (4)	32.2 ± 5.8	323.9 ± 245.0	8.8 ± 1.6	279.6 ± 201.1	454.9 ± 370.8	13.0 ± 14.0	21.6 ± 19.6	10.5 ± 12.6
(iii) Rice fields and animal hoof prints (4)	35.2 ± 3.2	633.1 ± 143.4	8.5 ± 1.1	520.6 ± 140.6	945.3 ± 265.2	10.0 ± 7.4	1.6 ± 1.9	3.1 ± 2.3
(iv) Swampy areas (10)	32.8 ± 4.7	529.1 ± 278.4	8.1 ± 0.8	428.9 ± 242.1	770.1 ± 409.1	10.0 ± 6.9	10.4 ± 8.6	4.1 ± 4.8
(v) Pond water reservoirs (3)	41.9 ± 0.8	990.0 ± 550.7	8.3 ± 0.0	809.2 ± 453.9	1336.0 ± 738.1	3.5 ± 2.8	0.9 ± 0.9	0.8 ± 0.7
(vi) Small ponds and water collections on the sides of the bridges (3)	28.7 ± 2.6	249.3 ± 103.9	8.5 ± 0.1	176.0 ± 58.8	335.7 ± 117.1	7.1 ± 12.3	9.3 ± 15.2	5.2 ± 8.9

In general, the association between mean larval and pupa densities of *Anopheles* and *Culex* were highest in breeding sites (category ii) comprising roadsides water collections and in road potholes (Table 4). This category had the highest pH and the second lowest salinity and conductivity values. The lowest larvae and pupae densities were found in pond water reservoirs (category v). This category had highest mean temperature, tds, salinity and conductivity. Surprisingly, except for temperature, which was the lowest, this site had the highest standard deviations of all physicochemical parameters. The mean values of these parameters remained consistently high even when data were log-transformed.

Table 5 shows results from GEE model based on lower and uppers percentiles of physicochemical parameters in different breeding sites on mosquito larvae and pupae (square root-transformed) densities. The type of breeding site was associated with the larvae and pupa densities wherein the univariate model, breeding site types such as rice fields and animal hoof prints as well as small pond water reservoirs had significantly lower densities of *Culex* larvae. However, in the multivariate analysis, *Anopheles* larval density was significantly lower in only small pond water reservoirs. None of the breeding sites was statistically significant for *Culex* larvae.

**Table 5:** Univariate and multivariate association between larvae densities with physiochemical parameters and breeding site-type

Parameter	Univariate		Multivariate	
	Coefficient (95% CI)	*P*-value	Coefficient (95% CI)	*P*-value
*Anopheles* larvae				
Breeding site type category				
(i) Rain water collections	1		1	
(ii) Road side water collections and road potholes	0.08 (-2.22 – -2.37)	0.947	-0.35 (-1.84–1.15)	0.650
(iii) Rice fields and animal hoof prints	-0.17 (-2.11–1.76)	0.860	-2.1 (-5.05–0.85)	0.162
(iv) Swampy areas	-0.19 (-1.65–1.26)	0.795	-0.36 (-1.86–1.13)	0.633
(v) Pond water reservoirs	-1.36 (-2.93–0.21)	0.089	-2.02 (–3.42 – -0.62)	0.005^a^
(vi) Small ponds and water collections on the sides of the bridges	-1.31 (-4.07–1.45)	0.351	0.001 (-3.00–3.00)	0.999
Salinity (ppm)	1.86 (0.59–3.12)	0.004^a^	2.55 (1.44–3.66)	< 0.001^a^
Temperature (°C)	0.06 (-1.40–1.52)	0.94		
Total dissolved solids (ppm)	1.35 (-0.01–2.70)	0.051		
Conductivity (μS)	1.62 (0.24–3.00)	0.022^a^		
pH	-0.45 (-1.26–0.35)	0.271		
*Culex* larvae				
Breeding site type category				
(i) Rain water collections	1		1	
(ii) Road side water collections and road potholes	0.66 (-2.14–3.45)	0.644	-0.232 (-2.52–2.06)	0.843
(iii) Rice fields and hoof prints	-2.59 (-4.33 – -0.85)	0.004^a^	-1.15 (-3.84–1.54)	0.402
(iv) Swampy areas	-0.72 (-2.59–1.16)	0.453	-1.39 (-3.38–0.59)	0.169
(v) Pond water reservoirs	-2.72 (-4.39–1.06)	0.001^a^	-1.57 (-4.34–1.19)	0.264
(vi) Small ponds and water collections on the sides of the bridges	-1.36 (-4.42–1.69)	0.382	-2.48 (-5.07–0.12)	0.062
Salinity (ppm)	0.57 (-0.22–2.37)	0.532		
Temperature (°C)	-1.75 (-3.36–0.12)	0.035^a^	-1.81 (-3.62–0.01)	0.051
Total dissolved solids (ppm)	-0.06 (-1.90–1.77)	0.95		
Conductivity (μS)	-0.01 (-1.93–1.91)	0.992		
pH	-0.74 (-1.42–0.06)	0.034^a^	-0.42 (-1.54–0.7)	0.459

^a^Statistically significant

In the univariate analysis, upper percentiles of salinity and conductivity were significantly associated with increased density of *Anopheles* larvae. The mean square root larval density was higher by 1.86 (95% CI: 0.59–3.12, *P* = 0.004) in upper percentiles of salinity compared to lower percentiles. For *Culex* larvae, temperature and pH were both associated with decrease in square root larval density by 1.75 (95% CI: 0.12–3.36, *P* = 0.035) and 0.74 (95% CI: 0.06–1.42, *P* = 0.034), respectively (Table 5).

Table 6 shows the GEE model based on lower and upper percentiles of physicochemical parameters in different breeding sites on pupae density (square root-transformed). Pupae density was significantly higher in breeding sites such as rice fields and animal hoof prints, swampy areas and small pond water reservoirs compared to rain water collections. Regarding the physicochemical parameters, the only temperature had statistically significant effect; and it was associated with a decrease in the square root of pupae density by 1.66 (95% CI: 0.75–2.57, *P* < 0.0001).

**Table 6:** Univariate and multivariate association between pupa densities with physiochemical parameters and breeding site-type

Parameter	Univariate		Multivariate	
	Coefficient (95% CI)	*P*-value	Coefficient (95% CI)	*P*-value
Breeding type category				
(i) Rain water collections	1		1	
(ii) Road side water collections and road potholes	0.39 (-1.3–2.09)	0.65	-0.39 (-2.71–1.93)	0.741
(iii) Rice field and animal hoof prints	-1.24 (-2.12 – -0.37)	0.005^a^	-0.98 (-1.85 – -0.12)	0.028^a^
(iv)) Swampy areas	-1.15 (-2.11 – -0.19)	0.018^a^	-1.28 (-2.06 – -0.50)	0.001^a^
(v) Pond water reservoirs	-1.89 (-2.68 – -1.09)	< 0.001^a^	-1.57 (-2.23 – -0.90)	< 0.001^a^
(vi) Small ponds and water collection on the sides of the bridges	0.75 (-0.04–1.53)	0.063	-0.07 (-0.43–0.30)	0.712
Physicochemical parameters				
Salinity (ppm)	-0.23 (-1.44–0.86)	0.618		
Temperature (°C)	-1.66 (-2.57 – -0.75)	< 0.001^a^	-0.69 (-1.44–0.06)	< 0.001^a^
Total dissolved solids (ppm)	-0.72 (-2.12–0.68)	0.312		
Conductivity (μS)	-0.64 (-1.94–0.66)	0.334		
pH	0.15 (-0.26–0.56)	0.481		

^a^Statistically significant

**Fig. 3:**
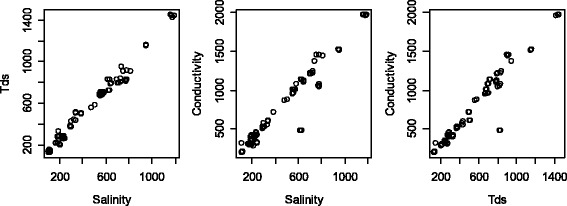
Scatter plots showing the relationship between total dissolved solids (tds), salinity and conductivity

In the multivariate model, tds and conductivity were excluded from the model due to their high colinearity with salinity (Fig. 3). The correlation coefficients between either of the two parameters were above 95%, which means that variation observed due to salinity could be explained by either of the two parameters. This model showed that the effect of the physicochemical parameters remained the same in exception of pH which turned out to be significant in the model as well as salinity which showed the slightly improved effect on *Anopheles* larvae density.

## Discussion

Our results indicate that presence of various physicochemical parameters in mosquito breeding sites at various levels has some influence on mosquito vector oviposition, survival and spatial distribution. Some physicochemical parameters appeared to significantly influence the larval density of individual mosquito species [30]. This study has also demonstrated that various levels of physicochemical parameters have effects on *Anopheles* and *Culex* larvae and pupae densities. Information on physicochemical characteristics of the breeding sites may have implication on vector distribution and disease transmission [9, 31]. This study has shown that both *Anopheles* and *Culex* larvae can co-exist in breeding sites despite their differences in breeding ecology. These findings concur with previous studies in western Kenya highlands under semi-field conditions [32] and field environments in northern Ethiopia [33]. There was a higher likelihood of finding *Anopheles* larvae over *Culex*. Similar findings were reported in Ghana, where *An. coluzzi* larvae have a higher tolerance level to faecal contaminated breeding sites than *Culex* larvae [34]. The difference in densities and the likelihood of finding species-specific larvae shows that the physicochemical parameters have some effects on larval growth [8, 12].

In the study area, *An. gambiae* (*s.l.*), *An. funestus* and *Cx. quinquefasciatus* were the main vectors of lymphatic filariasis [5, 35, 36] while *An. gambiae* (*s.l.*), *An. funestus* group are responsible for malaria transmission [6, 35]. PCR results showed that within the *An. gambiae* complex, only two sibling species identified namely *An. gambiae* (*s.s.*) and *An. arabiensis* but no *An. merus* was identified. A previous study conducted in the same area on vector species composition also did not find *An. merus* [3]. In addition, four sibling species were identified within the *An. funestus* group namely *An. funestus* (*s.s.*)*, An. rivulorum, An. leesoni* and *An. parensis.* These findings concur with a previous study conducted in the north-eastern Tanzania, which reveal the occurrence of the four-sibling species of the *An. funestus* group [2]. Among the sibling species identified *An. funestus* (*s.s.*) was the most common [2, 37].

The present study has shown that mosquito breeding sites varied from fresh to highly polluted habitats. Fresh mosquito breeding habitats are clean waters with little organic matter dissolved while highly polluted mosquito breeding habitats are sites with high levels of salinity, ammonium ions and organic pollution as described by Kudom [34] and Sattler et al. [38]. In the context of this study, highly polluted habitats are those with high tds, conductivity, salinity, temperature and pH. High concentrations of these parameters can be harmful to some species of mosquitoes [30, 39].

Physicochemical parameters survey in various mosquito breeding sites has revealed that the quality of water ranges from fresh to highly polluted [29], both with the presence of various densities of *Anopheles* and *Culex* mosquito larvae. The present study has demonstrated for the first time the occurrence of *Anopheles* in polluted habitats in rural settings of Tanzania. This was an unexpected finding especially in this rural setting for *An. gambiae* (*s.l.*), which usually breed in fresh water. Similar findings have been reported in previous studies conducted in the city of Dar es Salaam [38] and urban Lagos, Nigeria [39], but unlike our study, these studies were conducted in urban settings. This finding contradicts some previous findings, which reported that *Anopheles* mosquito breeds in fresh unpolluted water [6]. Unlike *Anopheles,* the *Culex* mosquito has been reported to breed in polluted waters such as septic tanks [40], pit latrine and soakage pits [41].

The occurrence of *Anopheles* larvae in breeding sites with high levels of salinity and conductivity suggests a gradual potential emergence of tolerance to these physicochemical parameters [2]. Nevertheless, the occurrence of high levels of physicochemical parameters such as conductivity, nitrate, sodium and ammonia in mosquito breeding sites are suspected to originate from the use of agricultural pesticides [9, 42, 43]. This tolerance could pose a serious threat to the success of various larvicidal products with similar chemical structure and activity with chemicals in the breeding sites. Moreover, emerged adult mosquito from such breeding sites are likely to be selected for inherent and acquired resistance to chemical products used for mosquito vector control even in the absence of prior exposure [10, 43, 44].

Mean densities of *Anopheles* larvae and *Culex* larvae and pupa, were highest in road side water collections and road potholes. Both species were collected in water with tds ranging from mineral springs to high contamination, salinity ranging from fresh water to polluted water and conductivity ranging from domestic to highly polluted water while pH was weak basic. This study has revealed that these mosquito species breed in a diverse range of habitats, contrary to current knowledge that *Anopheles* prefers to breed in fresh (unpolluted) clear water [6–8].

Mean larvae and pupae densities of *Anopheles* and *Culex* were highest in roadsides water collections and road potholes. These breeding sites were less permanent, small and resulting from human activities [45, 46] such as agriculture and infrastructural development. They also had the highest mean pH (above 7). These findings are in line with other reports which have revealed that *Anopheles gambiae* can breed in water with pH between 4 and 7.8 if there is sufficient aquatic food to consume [12]. Our findings are contradictory to those of Adebote et al. [47], which reported that *Anopheles* prefers low pH.

The lowest *Anopheles* and *Culex* larvae and pupae densities were recorded in pond water reservoirs, which were natural and permanent water bodies. In such kinds of larval habitats, the presence of predators and competitors could suppress mosquito larvae densities [45]. Pond water reservoirs had the highest mean temperature, tds, salinity and conductivity. These findings imply that the mentioned physicochemical properties may have been higher and beyond levels that could be tolerated by the identified mosquitoes. For instance, temperature above 30 °C affects growth and survival of *Anopheles* larvae because these are an enzyme-catalysed reaction and they decrease at high temperatures [8].

*Anopheles* larvae abundance was higher in waters with high levels of salinity and conductivity compared to *Culex.* These findings concur with a previous study conducted in northern Ethiopia [33] and in Accra, Ghana [10]. High levels of conductivity may be due to the application of agricultural pesticides and herbicides [9, 43]. Agricultural water runoff and sewage leaks are normally higher in nutrients such as chloride, phosphate and nitrate ions, therefore, leads to higher concentrations of dissolved solids which can influence conductivity [29]. Upper percentiles of salinity and conductivity significantly favoured *Anopheles* larvae compared to lower percentiles. Similar findings were also reported by Kweka and others in western Kenya highlands [31]. In this study, none of the physicochemical parameters was found to be significantly associated with the presence of *Culex.* These findings are contradictory to observations reported from Ethiopia [33] by Dejenie and others indicating that pH and conductivity were positively associated with the presence of *Culex.*

The upper percentiles of salinity and conductivity were significantly associated with increased density of *Anopheles* larvae. The mean square root of larval density was significantly higher by 1.86 in upper percentiles of salinity compared to lower percentiles. These findings are similar to a previous study conducted on the Kenyan coast on *An. merus* [30]. Unlike the present study, a study conducted in Nigeria reported that conductivity and tds appeared to have no influence on *Anopheles* larval density [8]. In the present study, upper percentiles of temperature were found to be associated with increased density of *Anopheles* larvae, although not statistically significant. This finding concurs with previous studies that, *Anopheles* larval abundance increases with increasing temperature from 28 to 32 °C [8, 10, 31, 48].

Unlike *Anopheles* larvae, for *Culex* larvae, temperature and pH were both associated with a decrease in larval density. Across all breeding sites, water mean pH ranged from 8.0 to 8.8. Breeding sites with high pH range are not ideal for mosquito breeding and survival due to free ammonia, which tends to increase with rising pH. Neutral pH between 6.8 and 7.2 is a preferred breeding site by mosquitoes. Therefore, outside this range mosquito eggs, larvae and pupae growth are reduced, and pH range below 4.5 or above 10, mortality occur [11]. The only temperature was found to be associated significantly with a decrease in pupae density by 1.66.

## Conclusion

The present study has found that the abundances *of Anopheles* and *Culex* larvae are affected by physicochemical parameters present in their breeding sites. However, the present study has revealed for the first time the occurrence of *Anopheles* larvae in polluted breeding sites in rural settings in Tanzania. In addition, the study has found both *Anopheles* and *Culex* mosquito larvae co-existing in breeding sites despite their differences in breeding ecology under natural conditions. These mosquitoes are vectors of malaria and lymphatic filariasis. This information is very useful in understanding the breeding behaviour of *Anopheles* mosquitoes in polluted habitats. The possible reasons for tolerance to a higher level of physicochemical parameters among *Anopheles* mosquitoes population need to be investigated.

## Abbreviations

CI: Confidence interval
CRERC: Ethics Review Committee
GEE: Generalised estimating equation
MRCC: Medical Research Coordinating Committee
NIMR: National Institute for Medical Research
OR: Odds ratio
ppm: Parts per million
SD: Standard deviation
tds: Total dissolved solids
TRAction: Translating Research into Action
USAID: United States Agency for International Development
